# HPV16-E7 Expression in Squamous Epithelium Creates a Local Immune Suppressive Environment via CCL2- and CCL5- Mediated Recruitment of Mast Cells

**DOI:** 10.1371/journal.ppat.1004466

**Published:** 2014-10-23

**Authors:** Anne-Sophie Bergot, Neill Ford, Graham R. Leggatt, James W. Wells, Ian H. Frazer, Michele A. Grimbaldeston

**Affiliations:** 1 The University of Queensland Diamantina Institute, Princess Alexandra Hospital, Brisbane, Queensland, Australia; 2 Division of Human Immunology, Centre for Cancer Biology, University of South Australia and SA Pathology, Adelaide, South Australia, Australia; Harvard Medical School, United States of America

## Abstract

Human Papillomavirus (HPV) 16 E7 protein promotes the transformation of HPV infected epithelium to malignancy. Here, we use a murine model in which the E7 protein of HPV16 is expressed as a transgene in epithelium to show that mast cells are recruited to the basal layer of E7-expressing epithelium, and that this recruitment is dependent on the epithelial hyperproliferation induced by E7 by inactivating Rb dependent cell cycle regulation. E7 induced epithelial hyperplasia is associated with increased epidermal secretion of CCL2 and CCL5 chemokines, which attract mast cells to the skin. Mast cells in E7 transgenic skin, in contrast to those in non-transgenic skin, exhibit degranulation. Notably, we found that resident mast cells in E7 transgenic skin cause local immune suppression as evidenced by tolerance of E7 transgenic skin grafts when mast cells are present compared to the rejection of mast cell-deficient E7 grafts in otherwise competent hosts. Thus, our findings suggest that mast cells, recruited towards CCL2 and CCL5 expressed by epithelium induced to proliferate by E7, may contribute to an immunosuppressive environment that enables the persistence of HPV E7 protein induced pre-cancerous lesions.

## Author Summary

Worldwide, around 50% of sexually active women are believed to become infected by Human papillomavirus type 16, the major cause of cervical cancer, and 2% will remain infected and therefore at lifetime risk of developing cancer. Why some women remain infected is unknown. Here we used a mouse engineered to express the HPV16 protein (E7) in skin, which drives development of pre-cancer lesions. This protein induces skin thickening, and the thickened skin releases molecules that attract mast cells. We show further that these cells locally suppress the function of the immune effector cells that can reject E7 expressing skin. We believe that targeting mast cells or impairing their attraction to the HPV infected tissue might therefore reduce the risk of cervical cancer for women infected with HPV16 by enabling them to clear their chronic infection.

## Introduction

Cervical and other anogenital cancers represent around 5% of all cancers, and are mostly due to infection of anogenital epithelium by one of more than 15 recognized “high risk” human papillomaviruses (HPV) [1]. Worldwide, ∼50% of sexually active women are believed to become infected by HPV16, the HPV genotype of highest risk, during their lifetime. Although most high risk HPV infections spontaneously regress, 1 to 2% of infected subjects develop persistent infection, which can progress to pre-cancerous lesions, and to cervical cancer if untreated [2].

Mast cells (MCs) are an important subset of immune cells, often considered as the first responders to opportunistic pathogens and allergens [3], [4]. Defined by the expression of two surface markers, the receptor for IgE (FcεRI) and the receptor for stem cell factor (SCF) c-Kit, MCs are distributed at various densities below epithelial surfaces that interface with the external environment such as in the skin, airways and gastrointestinal tract, where they are strategically placed to rapidly alert the inflammatory infantry when required. In HPV associated cancers, MCs occur in HPV induced premalignant CIN 2/3 lesions at twice the frequency observed in normal cervix [5]. Most CIN2/3 lesions, as well as cervical cancers, are known to be associated with infection of high-risk HPVs [6], [7]. In this study, we used HPV16-K14.E7 transgenic mice, in which sustained expression of HPV16 E7 protein in keratinocytes mimics pre-cancerous lesions caused by HPV infection, to address three important questions. Firstly, does HPV16 E7 expression recruit MCs toward the epithelium? Secondly, what mechanistic pathway is involved in this process? And thirdly, do MCs recruited juxtaposed to the epithelium by HPV16 E7 expression mediate local immune suppression?

We show that MCs are recruited preferentially to the basal layer of HPV16 E7 expressing epithelium, that this recruitment is dependent on HPV16 E7 induced epithelial hyperproliferation, and that it is likely MCs are recruited by a CCL2/CCL5 dependent mechanism. Using skin graft experiments in which HPV16 E7 expressing skin containing or lacking MCs is transferred onto wild-type C57BL/6 mice, in which we have previously demonstrated that graft rejection is CD8 T cell mediated, enhanced by IL-1 and γδ T cells, and hindered by passenger NKT cells in E7 transgenic skin, but not by regulatory T cells, [8]–[11], we now show that MCs make a key contribution to the immunosuppressive environment imposed by HPV16 E7-expression.

## Results

### HPV16-E7 expressing ear skin is hyperplastic and highly infiltrated by mast cells

**Figure 1:**
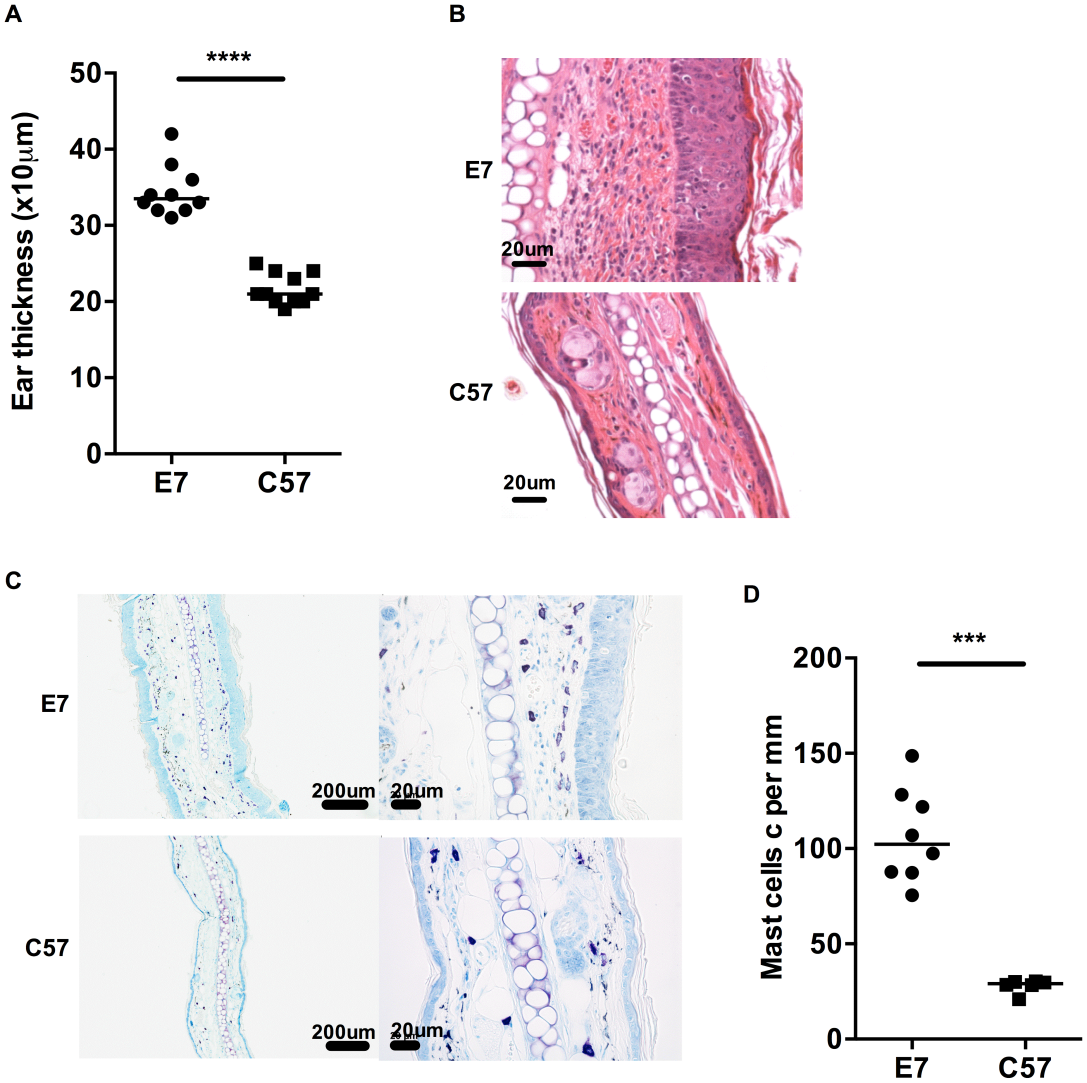
MCs are highly represented in E7 skin. A) Ear thicknesses were measured in naive age-matched E7 and C57 mice using a micrometer gauge (n = 10 and 11, respectively, ****p<0.0001). B) Representative images of E7 and C57 ear skin analyzed by H&E stain (scale bar  = 20 µm). C) Representative images of E7 and C57 ear skin analyzed by toluidine blue stain, which is specific for MCs (purple, scale bar  = 100 µm (left) and 20 µm (right)). D) MC numbers expressed per mm cartilage length (n = 8 E7 mice and n = 6 C57 mice, respectively; ***p<0.001).

K14.E7 transgenic mice (E7 mice) express the HPV16 E7 oncoprotein in epidermal keratinocytes under the control of the keratin 14 promoter. These mice are characterized by epidermal and dermal thickening, with an extensive dermal lymphoid infiltrate (**Fig. 1A, B**) consisting of an increased number of T cells [8], NKT cells [12] compared to C57BL/6 (C57) mice. Based on our previous findings that FoxP3^+^ T regulatory cells are not required to inhibit CD8 T cell function in HPV16 E7 skin graft tolerance [10], we hypothesized that other cells, along with NKTs, could interfere with HPV infection resolution. MCs represent one such population as there is important new evidence that they have anti-inflammatory functions in certain settings [13]–[16].

Mice transgenic for the entire genome of HPV16 demonstrate increased numbers of MCs in skin relative to non-transgenic animals [17]. To determine whether this was a consequence of E7 expression and associated epidermal hyperplasia, we assessed MC numbers in the dermis of E7 and wild-type skin by metachromatic toluidine blue staining (**Fig. 1C, D**). Wild-type skin showed detectable MCs (mean  = 27.96 cells per mm cartilage length) distributed predominantly at the dermal/hypodermal interface and sparsely in the upper dermis (**Fig. 1C** **; bottom panel**), whereas E7 skin exhibited significantly higher numbers of MCs tightly juxtaposed to the basal layer of the epidermis (mean  = 106.74 cells per mm cartilage length, p = 0.0007) (**Fig. 1C** **; upper panel and** **Fig. 1D**).

### HPV16-E7 driven hyperplasia is responsible for increased mast cell infiltration

**Figure 2:**
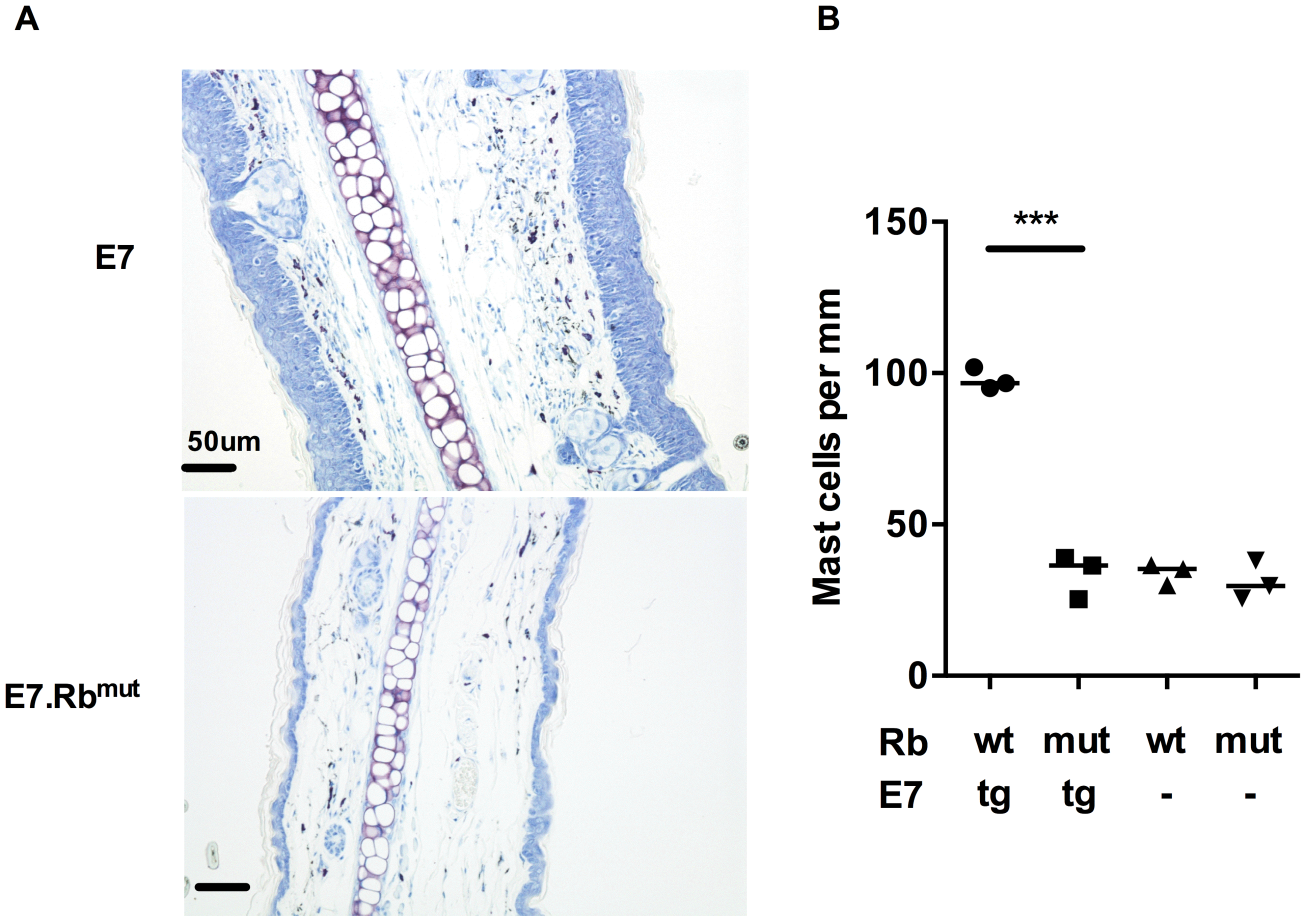
MC infiltration in ear skin is dependent on E7 hyperplasia. E7.Rb^mut^ ear skin is not hyperplasic and contains normal MC numbers. (A) Representative images of E7.Rb^wt^ and E7.Rb^mut^ ear skin by toluidine blue stain (original magnification ×400, scale  = 50 µm). (B) MC number per mm cartilage length (n = 3 in each group). ***p<0.001 by unpaired t-test for indicated comparison. Rb^wt^ are same as C57.

To determine whether E7 expression or the epithelial hyperplasia resulting from E7 expression is the primary cause of the increased numbers of MCs in E7 skin, we tested the impact of E7-induced hyperplasia on MC numbers. This was achieved using mice double transgenic for E7 and mutant Rb (E7.Rb^mut^) in which E7 cannot disrupt normal cell cycle regulation by segregating Rb [18]. We confirmed the lack of hyperplasia in E7.Rb^mut^ skin when compared to E7 mouse skin, which has unmodified Rb (**Fig. 2A** **bottom panel)**. The density of MCs in E7.Rb^mut^ skin was decreased to the level of C57 mouse skin and Rb^mut^ skin not transgenic for E7, as confirmed by analysis of MC numbers (**Fig. 2B**) (mean  = 97.88, 33.58, 33.93 and 31.10 MCs/mm cartilage length for E7.Rb^wt^, E7.Rb^mut^, Rb^wt^ and Rb^mut^, respectively, p = 0.0002). These findings strongly suggest that E7-mediated pRb inactivation is important for the recruitment of MCs to the epidermis.

### Mast cells are recruited to HPV16-E7 ear skin

The accumulation of MCs in hyperplastic skin associated with E7 expression might be due to prolonged survival, to local proliferation, or to increased recruitment [19]. To determine whether E7 induced epithelial hyperplasia induces MC proliferation, we stained serial ear pinna sections with toluidine blue to detect MCs and with PCNA to detect cell proliferation. Purple MCs and red PCNA stain did not co-localize (**Fig. S1A**) in wild-type or in E7 skin. To confirm this result, we cultured CFSE-labeled bone marrow-derived cultured MCs (BMCMCs) in medium alone, or with E7 and wild-type ear skin explant culture supernatants (all supplemented with IL-3) for 24, 48 and 72 h. Although BMCMCs proliferated in culture (**Fig. S1B**), their numbers were not different between cultures exposed to E7 or wild-type supernatants (**Fig. S1C**). Thus, there was no evidence that supernatants derived from hyperplastic E7 transgenic skin contained factors at levels high enough to induce MC proliferation.

**Figure 3:**
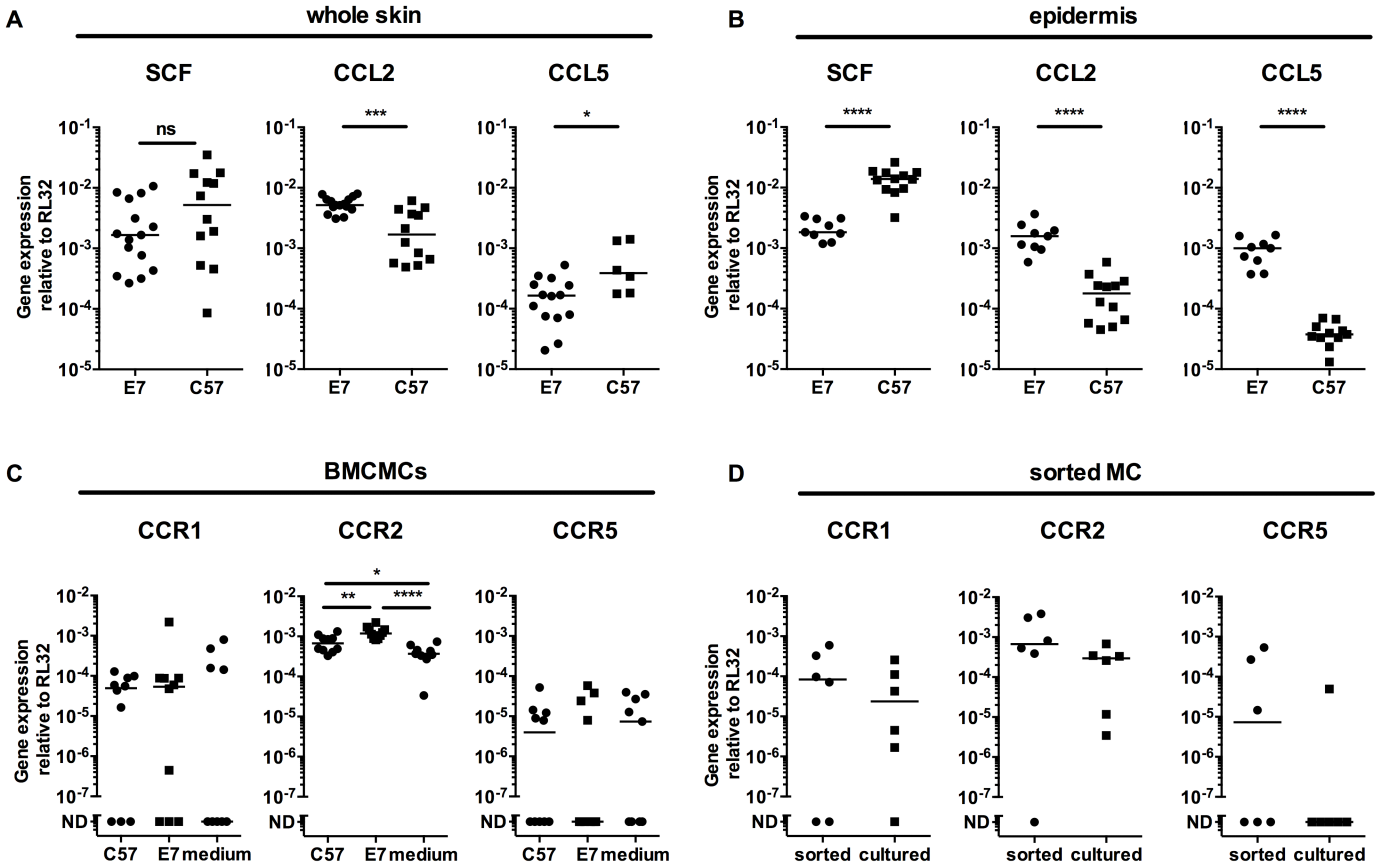
E7 epidermis produce CCL2 and CCL5 and MCs express the corresponding receptors. (A) C57 and E7 whole skin, or (B) C57 and E7 epidermis, were analyzed for SCF, CCL5 (Rantes) and CCL2 (MCP-1) gene expression. Data are combined from 2 independent experiments. CCR1 and CCR5 (two Rantes receptors) and CCR2 (MCP-1 receptor) gene expression in (C) BMCMCs after 72 h culture in E7 or C57 ear skin culture supernatant or medium (n = 10 different batches of BMCMCs; data are combined from 4 independent experiments. ND, not detectable) and (D) Mast cells sorted from E7 ear skin (n = 6 independent experiments, each with 4 or 5 mice pooled per treatment). Gene expression was performed by real time-PCR relative to the RL32 housekeeping gene. *p<0.05; **p<0.01; ***p<0.001; or ****p<0.0001 by unpaired t-test for indicated comparisons; ns  =  not significant.

To test whether E7 skin specifically recruited MCs, we examined the production of three relevant chemokines SCF, CCL2 (MCP-1) and CCL5 (Rantes). Although SCF mRNA expression was comparable between full thickness (dermis and epidermis) normal C57 versus E7 mouse skin, levels of CCL2 mRNA and CCL5 mRNA were significantly increased or decreased, respectively, in E7 skin (**Fig. 3A**). By contrast, E7 epidermis alone expressed lower levels of SCF mRNA (p<0.0001), and higher levels of both CCL2 and CCL5 than non-transgenic skin (p<0.0001) (**Fig. 3B**).

To determine whether MCs exposed to E7 skin might be induced to express the receptors for the chemokines upregulated in E7 skin, we co-cultured BMCMCs with ear skin explant supernatants for 72 h. BMCMCs exposed to E7 supernatants upregulated CCR2 mRNA (CCL2 receptor), and some but not all BMCMCs expressed CCR1 or CCR5 (CCL5 receptors) (**Fig. 3C**). Further, CCR2 mRNA was also expressed at higher levels by MCs sorted from E7 ear skin than by BMCMCs (**Fig. 3D**).

**Figure 4:**
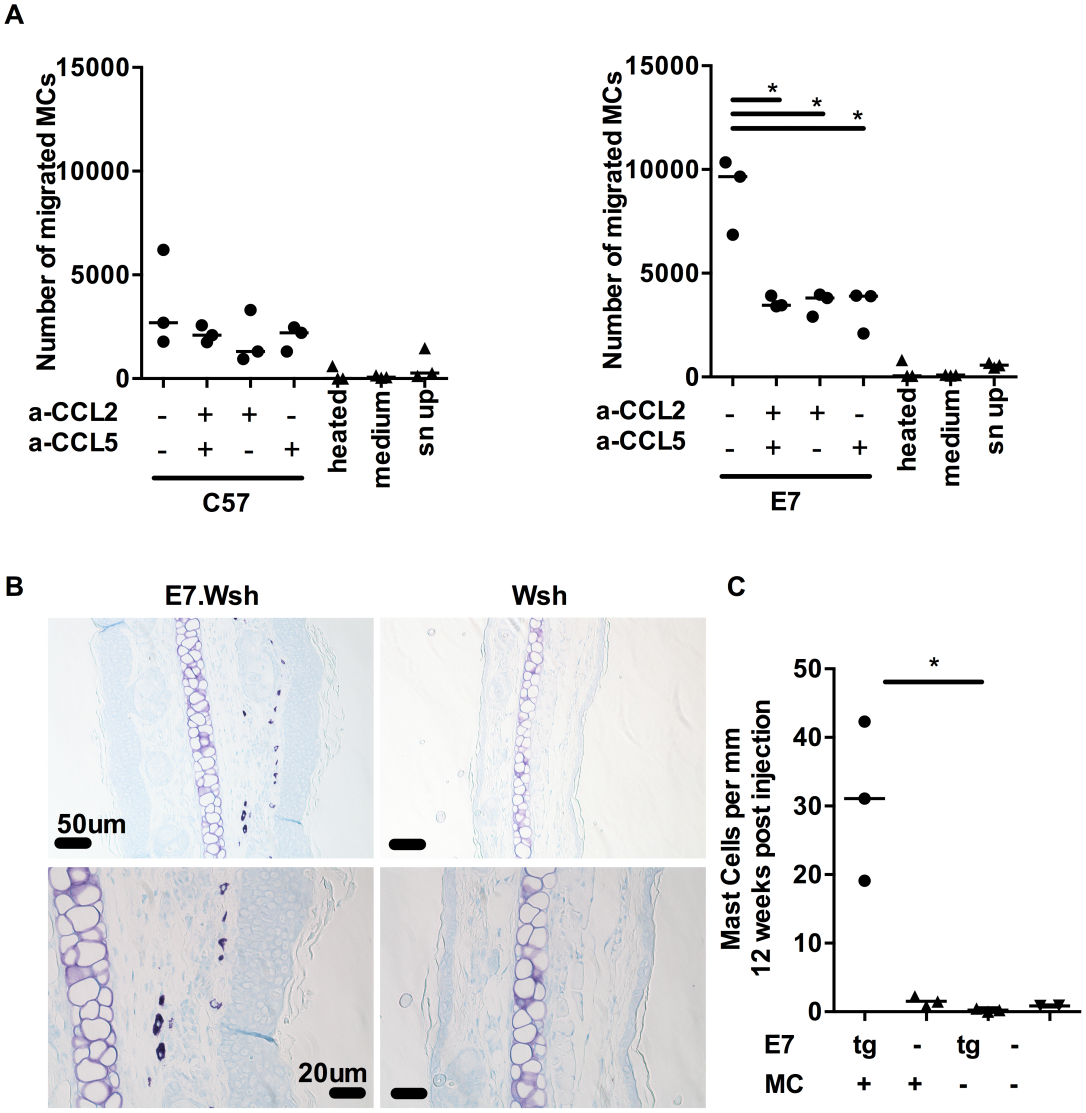
MCs migrate towards CCL2 and CCL5 and are recruited to E7 ear skin. (**A**) 5 µm transwell assays were used to determine the mast cells chemotaxis towards chemokines. Graphs represent the number of migrated FcεRIα^+^ cKit^+^ BMCMCs towards C57 skin explant supernatants (left) or E7 explant supernatants (right) in the presence of anti-CCL2 and/or anti-CCL5 blocking antibodies used at 10 µg/ml. 3 independent experiments (**B,C**) *Kit* ^W-sh/W-sh^ (Wsh) (n = 3) and E7.*Kit* ^W-sh/W-sh^ (E7.Wsh) (n = 3) mice were reconstituted with 1.4×10^7^ BMCMCs *i.v*. or left untreated (E7.Wsh (n = 3) and Wsh (n = 2)). 12 weeks later, mice were culled and MCs were identified in ear skin by (**B**) toluidine blue staining (**top**, scale bar  = 50 µm and **bottom**, scale bar  = 20 µm) and expressed as (**C**) MC number per mm cartilage length. *p<0.01, by unpaired t-test for all indicated comparisons.

To assess the migration capacity of MCs, we used BMCMCs in a migration assay. We first confirmed that BMCMCs migrate towards the direct ligand of cKit, SCF, as previously reported [20]. Then, we observed that BMCMCs migrate towards E7 ear skin explant supernatant, but not towards medium only or 98°C heated supernatant, suggesting that thermosensitive compounds attract BMCMCs (**Fig. 4A**). Moreover, BMCMCs do not migrate when the supernatant is added to the upper well chamber; demonstrating migration is due to chemotaxis rather than enhanced chemokinesis. Finally, we showed that this migration towards ear skin supernatant can be abrogated by blocking CCL2 and CCL5, together or alone (**Fig. 4A** **)**.

C57BL/6-*Kit* ^W-sh/W-sh^ mice have an inversion mutation that affects transcriptional regulatory elements upstream of the c-*kit* transcription start site on mouse chromosome 5 which impairs *Kit* function and results in a profound mast cell-deficiency in adult mice [21], [22]. To further establish *in vivo* that MCs are recruited preferentially to E7-expressing skin, we performed adoptive transfer *i.v.* of 1.4×10^7^ BMCMCs to *Kit* ^W-sh/W-sh^ and E7.*Kit* ^W-sh/W-sh^ mice. The recipient *Kit* ^W-sh/W-sh^ and E7.*Kit* ^W-sh/W-sh^ mice exhibited successful transfer of MC populations as evidenced by a substantial MC infiltration in the spleen of each mouse (**Fig. S2**) [21]. Notably, we observed preferential recruitment of the injected MCs to E7-expressing, as opposed to non-transgenic, ear skin tissues (**Fig. 4B, C**).

Altogether, these data suggest that BMCMCs are preferentially recruited to E7-expressing epidermis by a mechanism that is likely to involve the local production of CCL5 and CCL2.

### Mast cells are more degranulated in HPV16-E7 ear skin

Following the observation that BMCMCs were recruited to E7.*Kit* ^W-sh/W-sh^ ear skin, we also noticed their close contact with the basal layer of the epidermis in both the engrafted E7.*Kit* ^W-sh/W-sh^ mice and the E7 mice. Fully granulated MCs can be identified as dark purple stained cells using toluidine blue, while degranulated MCs are light purple stained with some exterior granules [23], [24]. In E7 skin, we observed that the closer MCs were to the keratinocytes, the more degranulated they appeared (**Fig. 1C** and **Fig. S3A**). Using a heparin stain, we assessed the degranulation status of MCs in E7 and C57 skin and we showed that E7 skin contained more degranulated MCs (**Fig. S3B**). Although the mechanism(s) underlying MC activation in this setting are yet to be fully elucidated, a potential candidate is endothelin-1 (ET-1) which can induce MC degranulation [25] and its expression is significantly elevated in E7 ear skin (**Fig. S4**). Thus, these data suggest that interaction of MCs with the basal epidermal layer in E7 skin, or with a factor secreted by the epidermis such as ET-1, can cause activation of MCs, with release of granule contents.

### Mast cells play a role in the immunosuppressive environment in HPV16 E7 skin

**Figure 5:**
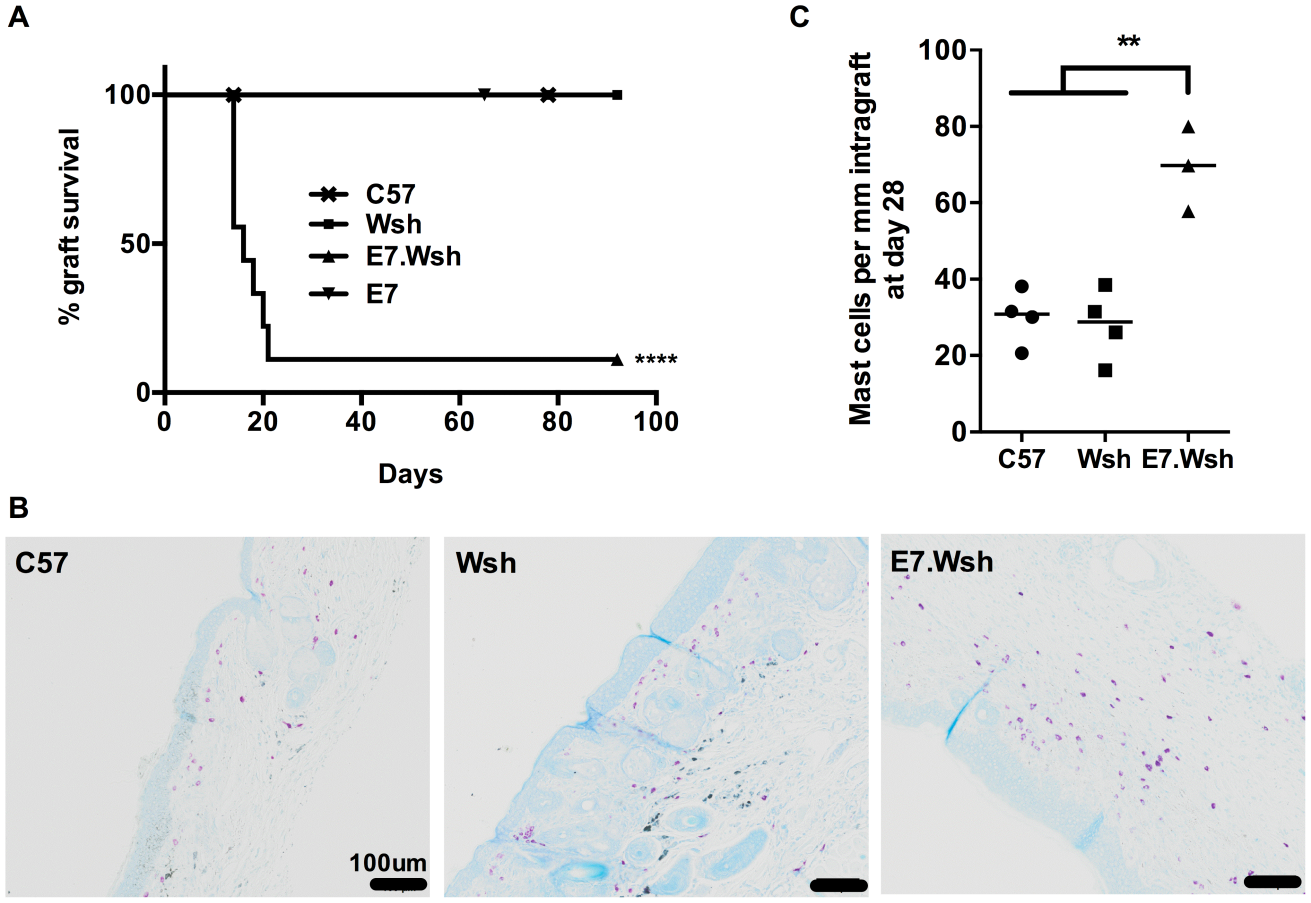
MCs are immunosuppressive in E7 environment. (A) C57 mice were double grafted with C57 syngeneic ear skin (n = 9), and either *Kit* ^W-sh/W-sh^ (Wsh) ear skin (n = 9) or E7. *Kit* ^W-sh/W-sh^ (E7.Wsh) ear skin (n = 9). Control E7 ear skin (n = 6) are tolerated. Kaplan-Meier survival curves show the median graft survival. ****p<0.0001 using a log-rank test. Data are combined from 3 independent experiments. (**B**) Representative graft histology of C57, Wsh and one remaining E7.Wsh graft at day 90 by toluidine blue staining (scale bar  = 100 µm). (**C**) MCs per mm of graft tissue at day 28 in C57 (n = 4), Wsh non-rejected (n = 4) and E7.Wsh rejected (n = 3) grafts. (**p<0.01, by unpaired t-test).

MCs can regulate local immune responses to tumors [17], [26] and allografts [27]–[29]. E7 skin grafted onto syngeneic, non-transgenic animals is not rejected [12]. To determine whether MCs contribute to the local immunoregulatory environment in this model, we grafted E7 skin with (E7) or without (E7.*Kit* ^W-sh/W-sh^) MCs onto syngeneic immunocompetent C57BL/6 recipients. E7 skin grafts without MCs were rejected within 3 weeks in 8 out of 9 mice, whilst E7 skin grafts with MCs, and non-transgenic skin with or without MCs, were not rejected (**Fig. 5A**). 28 days after grafting, host-derived MCs had repopulated the MC-deficient grafts, with the highest infiltration occurring within any remaining E7.*Kit* ^W-sh/W-sh^ skin (**Fig. 5B and 5C**), confirming the results of **Fig. 4**. These dataset demonstrate that MCs have a locally immunosuppressive effect on graft rejection, a regulatory function that might reduce CD8 T cell activity that we have previously shown to be necessary for graft rejection [8]–[11].

## Discussion

Infection of the cervix with high risk HPVs is necessary though not sufficient for the development of cervical cancer, and continued expression of HPV E6 and E7 non-structural proteins is the hallmark of HPV transformed epithelium [2]. Here we show, using a mouse with persisting epithelial expression of the E7 protein of HPV16, the high risk HPV most often associated with cervical cancer, that MCs are recruited to E7-expressing hyperplasic epithelium in high numbers, and that such recruitment is dependent on the ability of E7 to sequester Rb protein. We show further that degranulated MCs are found juxtaposed to the basal keratinocyte layer and are attracted to the skin by chemokines released by hyperproliferative epithelium, and that this accumulation might hinder, directly or indirectly, CD8 cytotoxic T cell mediated rejection of E7 expressing epithelium [8]–[11].

In mice transgenic for the whole HPV16 early gene region expressed from a K14 promoter, progression of epithelial hyperplasia and papillomatosis to dysplasia correlates with MC accumulation and activation [30], [31], but the mechanisms promoting the accumulation of MCs are poorly defined. Many chemoattractants are involved in the recruitment of MCs into tissues [32]. MCs are recruited to tumors by tumor-derived SCF [33], engaging a signaling pathway for MC differentiation, migration, maturation and survival [34]. In virus associated tumors, additional chemokines, and their respective receptors expressed on the cell surface of circulating bone marrow-derived progenitors [35] or resident mature MCs, are likely to provide migratory or proliferative signals. Coussens et al. [17], [36], [37] observed, in a HPV16 transgenic mouse in which the entire early gene region of HPV16 was expressed in the skin under the K14 promoter, that there was infiltration of MCs and increased angiogenesis in association with keratinocyte proliferation and increased skin thickness. To study the contribution of MCs, Coussens et al. attempted to use c-*kit* mutant *Kit* ^W/W-v^ mice that exhibit a profound loss of c-*kit* activity, resulting in a systemic MC and basophil deficiency, as well as other phenotypic abnormalities including anemia, neutropenia and sterility [14]. A single *Kit* ^W/W-v^ MC-deficient mouse expressing HPV16 early genes was the sole survivor of 89 *Kit* ^W/W-v^ MC-deficient pups among 700 littermates, precluding analysis of the role of MCs in HPV associated pathology. To establish a role for MCs in conferring local immunosuppression or promoting local pathology in HPV transformed epithelium, we therefore studied a mouse which expresses HPV16 E7, the single HPV early gene most relevant to cervical epithelial progression to cancer, from a keratin 14 promoter. We used a C57BL/6-*Kit* ^W-sh/W-sh^ mouse, in which an inversion mutation of the transcriptional regulatory elements disrupts *Kit* transcription leading, as in the *Kit* ^W/W-v^ mouse, to a profound systemic MC deficiency. This *Kit* ^W-sh/W-sh^ MC-deficient strain exhibits a neutrophilia [22], [38] rather than the neutropenia of *Kit* ^W/W-v^ mice, and otherwise exhibits a much reduced range of phenotypic abnormalities [21], [22], [38]. When crossed with our HPV16 E7 mice, the C57BL/6-*Kit* ^W-sh/W-sh^ mice produce viable E7 transgenic and MC-deficient offspring.

In our MC competent HPV16 E7 mice, we confirmed Coussens' observation of the accumulation of MCs in the dermis [17], juxtaposed to the basal layer of the epidermis where E7 is expressed. We therefore proceeded to establish the mechanism for accumulation of MCs at that site. E7 expressed under the K14 promoter interacts with many proteins and also binds its main target Rb inhibiting the sequestration of E2F family proteins, and thus driving keratinocyte proliferation [8], [18], [21], [39]. A mutation of Rb is recognized that hinders binding of E7 but not of E2F proteins [8]. E7.Rb^mut^ mice, expressing E7 and this mutated Rb, do not show the epithelial hyperplasia associated with E7 transgenic mice. We observed no increase in MCs in E7.Rb^mut^ mice, confirming that MC infiltration of the basal epidermis is associated with HPV16 E7 induced epithelial hyperproliferation.

We have previously observed that IFNγ is expressed in E7 transgenic mouse skin [12], [40], and therefore chemokines expressing an IFN regulatory factor-1 response element in their non-coding gene regions, such as CCL2 and CCL5 [41], [42], represent potential MC chemoattractants to E7 skin. We show here that MCs migrate towards HPV16 E7-expressing ear skin explant cultures, and that migration towards the supernatant of HPV16 E7-expressing ear skin cultures can be blocked by neutralizing CCL2 and CCL5, which confirmed a role for these chemokines in recruiting MCs to HPV16 infected epithelium. Adoptive transfer of BMCMCs repopulated the MC population in the ear skin of MC-deficient HPV16 E7. *Kit* ^W-sh/W-sh^ mice but not MC-deficient *Kit* ^W-sh/W-sh^ mice, demonstrating that expression of HPV16 E7 induces MC migration. High levels of CCL2 and CCL5 transcription associated with HPV16 E7 expression in skin are consistent with the hypothesis that expression of these chemokines, induced by E7, accounts for the MC infiltrate in E7 transgenic mouse skin. To further decipher the role of Rb and epithelial hyperproliferation in MC chemotaxis, future experiments will determine whether CCL2 and CCL5 levels are concomitantly decreased in Rb^mut^/PV16-K14.E7 Tg mouse epidermis.

As shown in Figure 1 and Supplemental Figure 3, those MCs recruited to E7 skin appear more degranulated. Although the mechanisms underlying MC degranulation at the interface with HPV16 E7 epithelium are yet to be fully understood, production of ET-1 by the virus affected epithelium [43] or by the surrounding microenvironment in E7 transgenic mouse skin (**Fig. S4**), and the ability of this peptide to promote tumor invasion [44] as well as induce MC degranulation [25] represents a potential means of MC activation in our current study. MC degranulation typically involves release of pre-formed and stored granule associated mediators [14], including histamine which can contribute to systemic immunosuppression in response to UVB-irradiation of the skin [45], and the tryptase mMCP6 which can actively deplete the local environment of IL-6 to maintain skin allograft tolerance [46]. The activated state of MCs in HPV16 E7 skin also indicates that MCs might be releasing *de novo* synthesized mediators. We have shown that MC-derived IL-10 can curtail inflammation associated with certain settings of allergic contact dermatitis and low-dose UVB irradiation of the skin [15]. More recently, other immunoregulatory roles for MC-IL-10 have emerged, including an ability to reduce graft-versus-host-disease independently of Tregs during hematopoietic cell transplantation [16], [47] and a capacity to drive tolerance in chronic bacterial infection by suppressing humoral and cell-mediated immunity [13]. However, while evidence from such studies supports the notion that MC-IL-10, histamine or mMCP6 might be involved in regulating immune responses in the HPV-infected microenvironment, further work is required to determine which MC-derived mediators specifically orchestrate local immune suppression in this setting.

MCs are involved in many pathologies and a role in cancer has been indicated (reviewed in [48], and [26], [49], [50]). The accumulation of MCs in the vicinity of tumor tissue strongly correlates with poor prognosis in many aggressive cancers, including gastrointestinal [51], [52] and pancreatic cancers [53] in humans, and in mice. MCs can promote angiogenesis, tumor invasion, immune suppression, and the recruitment of other immune cells including regulatory T cells [29]. However, the role of MCs in cervical cancer associated with HPV infection is largely unknown, but in such a setting it is possible that MCs promote persistence of infection by contributing to an immunosuppressive microenvironment. MC prevalence at different anatomical sites is under genetic control and can be influenced by extrinsic factors (e.g. extent of sun exposure of the skin) [54]. Thus MC heterogeneity in prevalence, as well as inter-individual differences in the microenvironments in which the MCs reside might be factors that contribute to allowing persistence of HPV infection, and hence increased risk of cancer, in only 2% of those infected. Persisting HPV infection is not resolved by the current preventive vaccines [55], [56], and new therapeutic strategies are needed to treat the many women at risk of cervical cancer through persisting HPV infection [57]. A specific immunotherapy against E6 and/or E7 remains elusive [2], [58], [59], though whether HPV infection directly suppresses aspects of the host immune response is largely unknown [60]. Vaccine immunotherapy against an HPV16 E7 expressing non-small cell lung cancer (NSCLC) line has been shown to be more effective with an anti-CCL2 blocking antibody [61]. Taken together, our data suggest that HPV16 E7-expression in the epithelium recruits MCs, which like tumor associated macrophages [62] and myeloid suppressor cells [63] in other malignant settings, appear to exhibit an immunosuppressive function in the E7-influenced microenvironment. Thus, it is plausible that CCL2 and/or CCL5 blockade might reduce such immunosuppression and facilitate immunotherapy of HPV associated cancers.

## Materials and Methods

### Mice

C57BL/6 mice (C57) were obtained from the Animal Resources Centre (ARC, Perth, Australia). HPV16 K14.E7 transgenic C57BL/6 mice (E7 mice), in which E7 oncoprotein is expressed under the K14 promoter were maintained locally at the Princess Alexandra Hospital Biological Research Facility (BRF, Brisbane, QLD, Australia) under specific pathogen-free conditions. Rb^DLXCXE^ (Rb^mut^) mice and HPV16 K14.E7x-Rb^DLXCXE^ (E7.Rb^mut^) mice on a mixed 129/FVB/C57 background have been previously described [8], [18] and were bred at the McArdle Laboratory Cancer Center Animal Care Facility, USA, and generously provided by PF Lambert lab, Madison, Wisconsin, USA. Genetically c-*kit* mutant mast cell-deficient C57BL/6-*Kit* ^W-sh/+^ mice backcrossed with C57BL/6J mice for 14 generations were used as breeding pairs to produce mast cell-deficient B6.*Kit* ^W-sh/W-sh^ mice and were maintained at the IMVS Animal Facility (Centre for Cancer Biology, Adelaide, SA, Australia [21], [64]). B6-*Kit* ^W-sh/W-sh^ mice were crossed with E7 mice to obtain mast cell-deficient mice expressing the HPV16-E7 oncoprotein (E7.*Kit* ^W-sh/W-sh^ mice). All mice were sex matched for all experiments and were used at 10 to 16 weeks of age. Experiments were performed in compliance with the ethical guidelines of the National Health and Medical Research Council of Australia, with approval from the IMVS Animal Ethics Committee and the University of Queensland Animal Ethics Committee.

Ear thickness was measured with a micrometer gauge (Ozaki MFG) on anesthetized mice.

### Mast cell isolation from ear skin

Ears were harvested and separated into dorsal and ventral halves using forceps. For epidermal removal, the skin was incubated epidermis-down in 1.2 mg/ml Dispase II (Roche) at 37°C. After an hour, the epidermal layer was peeled off the dermis. To release cells, skin were torn into small fragments and digested for 1 h in 1 mg/ml collagenase D, 0.5 mg/ml type 2 hyaluronidase and 20 ug/ml Dnase 1 (all from Roche) at 37°C. Tissues were passed through a cell strainer and washed in PBS containing 3% FBS. Isolated cells were then stained for flow cytometry or cell sorting using anti-CD3 (clone 2C11, 1.0 µg/ml), anti-CD45R/B220 (clone RA3-6B2, 1.0 µg/ml), and anti-CD117 (cKit clone 2B8, 1.25 µg/ml) antibodies from BD Pharmingen, and anti-CD45.2 (clone 104, 0.5 µg/ml), anti-FcεRIα (clone MAR-1, 0.5 µg/ml) antibodies and streptavidin PE (0.4 µg/ml) from eBioscience, and anti-CD11c (clone N418, 2.5 µg/ml) from BioLegend. MCs were gated as CD45.2^+^, CD3^−^, B220^−^, CD11c^−^, cKit^+^ and FcεR1α^+^. For mRNA isolation, sorted MCs were directly collected into lysis buffer (Bioline ISOLATE II RNA Micro Kit).

### BM-derived cultured mast cells

As previously described [15], [64], bone marrow cells were collected from femurs and tibiae and cultured in DMEM supplemented with 10% Fetal Calf Serum and a source of mouse IL-3 which is necessary for MC differentiation and proliferation (i.e. 20% WEHI-3 conditioned medium supplemented with recombinant mouse IL-3 (R&D Systems) to consistently achieve a total of 3–4 ng/mL IL-3). After 5 to 6 weeks >95% of the cells were identified as MCs by May-Grunwald-Giemsa staining histologically or by flow cytometry using anti-CD45.2^+^, cKit^+^ and FcεRIα^+^ staining.

### Adoptive transfer of BMCMCs

5 to 6 week old BMCMCs were washed twice in PBS and 1.4×10^7^ cells injected *i.v.* into *Kit* ^W-sh/W-sh^ and E7. *Kit* ^W-sh/W-sh^ mice. 12 weeks after BMCMC transfer into the mice, ear skin and spleen were collected to confirm the presence of MCs in these tissues by toluidine blue staining and histological analysis, as previously described [21].

### Ear skin explant culture

Ears were collected from C57 or E7 mice on ice, split into halves, and placed dermis side down in complete WEHI-conditioned medium at 37°C. Medium was replaced after 1 h and again after 3 h with 600 µl of fresh conditioned medium, to reduce cell-death related release of cytokines and danger signals. Ear explants supernatants were collected 20 h later and stored at −80 degrees until use.

For BMCMC culture with ear skin explant supernatants, 5×10^5^ BMCMCs were first labelled with 2.5 µM CFSE for 15 min at 37°C, and then washed twice with PBS. BMCMCs were then seeded in 24 well plates in WEHI-conditioned medium and ear skin explant supernatant (1∶1) for 4, 24, 48 or 72 h, following which cells were collected for mRNA extraction. CFSE dilution was analyzed by flow cytometry within cKit^+^ FcεRIα^+^ double-positive cells. Samples with analyses below the assay detection level were assigned a value of not detected (ND) for display and statistical analysis.

### Migration assay

Transwell migration assays were performed using 5 µm pore size Transwell inserts (Corning, NY). 2×10^5^ 3–6 week old BMCMCs were placed in the top chamber in 100 ul of medium without IL-3. Recombinant mouse SCF (RnD Systems) at 0–100 ng/ml or E7/C57 ear skin explant culture supernatant without IL-3 were placed in the bottom chamber. When indicated, anti-CCL2/Rantes (clone 53405) or anti-CCL5/JE/MCP-1 (clone 123616) blocking antibodies from RnD Systems were added in the bottom chamber at 10 µg/ml and the plate was then incubated at 37°C. Four hours later, cells were collected from the bottom chamber and counted by trypan blue exclusion on a hemocytometer, and phenotyped by flow cytometry for anti-CD45.2, anti-FcεRIα and anti-cKit expression.

### Ear skin grafting

Donor ear skin was grafted onto recipient flanks as previously described [12]. Briefly, dorsal and ventral surfaces of ear skin from transgenic mice were placed onto the thoracic flank region of an anesthetized C57BL/6 recipient. Grafts were held in place with antibiotic-permeated gauze (Bactigras; Smith and Nephew, London, U.K.) and bandaged with micropore tape and Flex-wrap (Lyppard, Queensland, Australia). After 7 days, bandages were removed and grafts were monitored three times a week for 4 weeks or longer. Graft rejection was assessed by a loss of distinct border and signs of ulceration and/or necrosis to >80% of the graft area.

### Histology on ear skin tissues

Mice were culled by CO_2_ inhalation and samples of ear pinnae were fixed in 4% formalin. Samples were coded using a serial number, so the evaluator was not aware of their identity and sent to the histology facility to be embedded in paraffin (ensuring a cross-sectional orientation) and cut as 4–6 µm sections. Sections were then stained with toluidine blue, pH 1, for the detection of mast cells (purple), with hematoxylin/eosin, alcian blue-safranin-O or with Proliferating Cell Nuclear Antigen (PCNA, Sigma). Images of coded samples were taken with a 20× microscope objective (Nikon Brightfield, final magnification, ×200). Field lengths (µm) were determined using NIS-Element software (Nikon). Mast cells were counted manually by image analysis using NIS-Element on 4 to 10 consecutive fixed fields of view along the entire length of ear skin and calculated per mm cartilage length.

### mRNA extraction and semi-quantitative real-time PCR

At collection, samples were snap-frozen in dry ice and stored at −80°C until mRNA extraction. Ear skin samples were then lysed in RNase-free microtubes using Trizol (Sigma) and an IKA T10 Ultra-Turrax homogenizer, and incubated for 5 min at RT. Total RNA extraction was performed as per manufacturer's recommendations. Briefly, 0.2 ml of mRNA-grade chloroform was mixed with each sample and incubated for 2–3 min. Samples were centrifuged 12000 rpm for 15 min at 4°C. The aqueous, colorless phase containing RNA was then collected without disturbing the white interphase and transferred into a fresh tube. RNA was precipitated using cold 100% isopropanol (vol/vol), incubation for 10 min at RT and centrifugation 12000 rpm for 10 min at 4°C. The RNA pellet was washed twice in 75% ethanol and air-dried for 10–15 min before being dissolved in 10 µl of RNase/Dnase free water at 55°C. Genomic DNA was digested using the Qiagen RNase-free DNase kit (DNase kit; #79254). RNAs were then quantified at 260/280 ratio by nanodrop spectrophotometry. RNAs were stored at −80°C until used for retrotranscription. For cell-sorted MCs, the Isolate II RNA Micro kit (Bioline) was used following manufacturer's instructions.

For reverse transcription, 500 ng of RNA was combined with 25 mM MgCl_2_, 25 mM dNTPs, oligoDT, RNase inhibitor, and MuLV Taq polymerase in buffer (all from Applied Biosystems) for 25 min at 25°C, 60 min at 42°C and 5 min at 95°C. cDNAs were stored at −20°C until used for PCR.

For semi-quantitative Real-Time PCR, samples were amplified using a Sybr premix Taq II (TAKARA) following the manufacturer's instructions. The amplification program was run on a ABI7900 (Applied Biosystems) - 1×30 sec at 95°C, 45× (5 sec 95°C and 30 sec at 60°C), followed by a dissociation stage (15 sec at 95°C, 60 sec at 60°C, 15 sec at 95°C). For the detection of ET-1, the following cycling conditions were performed: 1×15 min at 95°C, 45× (15 sec 95°C, 15 sec at 55°C, 20 sec at 72°C), followed by a hold of 30 sec at 72°C. Primers were designed using IDT (Integrated DNA Technologies, www.idtdna.com) (**Table S1**).

### Statistics

A non-parametric Mann-Whitney t-test or unpaired t-test were used as indicated for assessment of differences between groups. A Log-rank (Mantel-Cox) test was used to compare survival curves. Differences were considered to be significant when the p value was less than 0.05. Prism (GraphPad Software, La Jolla, CA) software was used to prepare graphs and for statistical analysis.

## Supporting Information

Figure S1 **MCs do not proliferate in E7 ear pinnae.** (**A**) Representative images of PCNA or toluidine blue stained C57BL/6-WT (C57) and E7 ears (scale bar  = 20 µm). Arrow heads indicate MCs in both PCNA and toluidine blue sections. (**B**) E7 ear explant supernatant does not induce increased BMCMC proliferation. CFSE-labelled BMCMCs were cultured in E7 or C57 ear skin culture supernatant or medium only for 24, 48 or 72 h and analyzed by flow cytometry for CFSE dilution. Data are representative of 2 independent experiments. (**C**) After 72 h, live and dead BMCMCs were counted (n = 10 different batches of supernatants per group, 4 different BMDMCs batches). Data expressed as mean + SEM and no significant differences were observed between the groups using a non-parametric Mann-Whitney statistical test.
(TIFF)

Figure S2 **Evidence that MC populations successfully engraft in recipient** ***Kit*** **^W-sh/W-sh^ and E7.** ***Kit*** **^W-sh/W-sh^ mice.** MCs (purple cells) identified in toluidine blue stained spleen sections of C57 (n = 1), MC-deficient *Kit* ^W-sh/W-sh^ (Wsh) (n = 1), and in *Kit* ^W-sh/W-sh^ (Wsh) (n = 3) or E7.*Kit* ^W-sh/W-sh^ (E7.Wsh) (n = 3) mice 12 weeks after i.v. administration of 1.4×10^7^ wild-type BMCMCs. (**A**) MC number per mm^2^; and (**B-D**) representative histological images of spleen sections from (**B**) MC-deficient *Kit* ^W-sh/W-sh^ (Wsh) and (**C–D**) BMCMC-engrafted E7.*Kit* ^W-sh/W-sh^ mice. **B,C**, scale bar  = 1 mm and **D**, scale bar  = 100 µm. Data expressed as mean + SEM.
(TIFF)

Figure S3 **MCs are more degranulated in E7 ear pinnae.** (**A**) Representative image of safranin stained MCs in an E7 mouse ear pinna (scale bar  = 10 µm). Degranulation of MCs indicated as non-degranulated (none), partial or high. e, epidermis; d, dermis; c, cartilage (**B**) Extent of MC degranulation expressed as the percent of the total MC counted in ear skin sections of a total of 2.8 to 3.3 mm cartilage length. Data expressed as mean + SEM; n = 3 mice each of C57BL/6- WT or E7 genotype). *p<0.05; or ***p<0.001 for indicated comparisons by an unpaired Student's t-test with Welch's correction.
(TIFF)

Figure S4 **ET-1 gene transcript is upregulated in E7 ear pinnae.** C57 and E7 whole mouse skin were analyzed for ET-1 mRNA. Gene expression was performed by real time-PCR relative to the RL32 housekeeping gene. Mice: 10–14 weeks of age; data expressed as mean + SEM; n = 12 mice/group *p<0.05 by unpaired t-test.
(TIFF)

Table S1 **Primer sequences.** Gene names, NCBI IDs and forward and reverse sequences of the genes analysed in this study.
(DOCX)
