# Supplementary material for: HPV16-E7 Expression in Squamous Epithelium Creates a Local Immune Suppressive Environment via CCL2- and CCL5- Mediated Recruitment of Mast Cells
Source: PLoS Pathog. 2014 Oct 23;10(10):e1004466. doi: 10.1371/journal.ppat.1004466 (PMC4207828; doi:10.1371/journal.ppat.1004466)
Supplement: Table S1 — Primer sequences. Gene names, NCBI IDs and forward and reverse sequences of the genes analysed in this study. (DOCX) [file ppat.1004466.s005.docx]

**HPV16-E7 Expression in Squamous Epithelium Creates a Local Immune Suppressive Environment via CCL2- and CCL5- Mediated Recruitment of Mast Cells**

Anne-Sophie Bergot^1^, Neill Ford^1^, Graham R. Leggatt^1^, James W. Wells^1*^, Ian H. Frazer^1*^, Michele A. Grimbaldeston^2*^

^1^The University of Queensland Diamantina Institute, Princess Alexandra Hospital, Brisbane, Qld, Australia;

^2^Division of Human Immunology, Centre for Cancer Biology, University of South Australia and SA Pathology, South Australia, Australia

* These authors share senior authorship of this work

**Supporting Table 1.**

| **Gene** | **NCBI ID** | **Forward sequence** | **Reverse Sequence** |
| --- | --- | --- | --- |
| **RPL32** | NM_172086 | AAGCGAAACTGGCGGAAAC | TAACCGATGTTGGGCATCAG |
| **SCF** | NM_013598 | TCAAGAGGTGTAATTGTGGACG | GGGTAGCAAGAACAGGTAAGG |
| **CCL2** | NM_011333 | GTCCCTGTCATGCTTCTGG | GCTCTCCAGCCTACTCATTG |
| **CCL5** | NM_013653 | GGGTACCATGAAGATCTCTGC | TCTAGGGAGAGGTAGGCAAAG |
| **CCR1** | NM_009912 | GTTGGGACCTTGAACCTTGA | TTTGCTGAGGAACTGGTCAG |
| **CCR2** | NM_009915 | GCCATCATAAAGGAGCCATACC | GGTGAATCCAATGCCCTCTTC |
| **CCR5** | NM_009917 | TGTACAGCTCTCCTAGCCAG | TCGGAACTGACCCTTGAAAATC |
